# Neurophysiology of empathy and its relationship to early development: Evidence from Colombian mothers

**DOI:** 10.1192/j.eurpsy.2026.11763

**Published:** 2026-07-31

**Authors:** S. Avila-Jimenez, M. Jiménez - Martinez

**Affiliations:** Boyaca, Universidad Pedagogica Y Tecnologica De Colombia, Tunja, Colombia

## Abstract

**Introduction:**

Research shows that many of the neural circuits responsible for executing an action are also activated when that action is observed or mentally represented in another person (Rizzolatti and Craighero, 2004). In this study, empathy is assessed using two measures: the first is a subjective measure provided by the Interpersonal Reactivity Index (Davis, 1980), a self-report with dimensions of perspective taking, fantasy, empathic concern, and personal distress. The second corresponds to objective measurements taken through psychophysiological measurements of the zygomatic and corrugator muscles using Power Lab while mothers observe IAPS images (Lange, 1995)

**Objectives:**

To examine the relationship between maternal empathy and child development in children aged 1 to 36 months in the Colombian population, based on the results of the Ages and Stages Questionnaire, third edition (ASQ-3).

**Methods:**

This study adopts a quantitative approach, using a cross-sectional correlational design to examine the relationships between variables at a given point in time. The sample consisted of 100 mothers residing in Tunja, aged between 18 and 44 years (M = 28.8), who had previously participated with their babies in the assessment of child development through the application of the ASQ-3. Non-probability convenience sampling was used.

**Results:**

Within the IRI, the subscale that showed a statistically significant difference was “Empathic Concern,” in which mothers with optimally developing babies tend to experience higher levels of compassion, concern, and affection toward the suffering of others, which is directly linked to their ability to develop empathetic responses toward others, similarly, it shows correlation to an emotional involvement that triggers behaviors of helping others. The variable number of children and age of the mother in relation to the zygomatic response obtained a statistically significant negative correlation, meaning that the more children and the older the mother, the lower the zygomatic muscle response and, therefore, the lower the emotional empathy

**Conclusions:**

The results provide empirical evidence combining psychological and physiological measures in understanding the relationship between maternal empathy variables and child development.

Davis, M. (1980). A multidimensional Approach to Individual Differen- ces in Empathy. *JSAS Catalog of Selected Documents in Psychology*, 10, 85. https://www.uv.es/friasnav/Davis_1980.pdf

Lang, P.. (1995).The emotion probe: Studies of motivation and attention. *American psychologist*, 50(5), 372. https://doi.org/10.1037/0003-066X.50.5.372

Rizzolatti, G., & Craighero, L. (2004). The mirror-neuron system. Annu. *Rev. Neuroscience.*, 27, 169-192. https://doi.org/10.1146/annurev.neuro.27.070203.144230

**Disclosure of Interest:**

None Declared

